# 3-year-data of combined navigated laser photocoagulation (Navilas) and intravitreal ranibizumab compared to ranibizumab monotherapy in DME patients

**DOI:** 10.1371/journal.pone.0202483

**Published:** 2018-08-23

**Authors:** Tina Rike Herold, Julian Langer, Efstathios Vounotrypidis, Marcus Kernt, Raffael Liegl, Siegfried G. Priglinger

**Affiliations:** 1 Department of Ophthalmology, University Hospital, LMU, Munich, Germany; 2 Forstenrieder Allee 59, Munich, Germany; LV Prasad Eye Institute, INDIA

## Abstract

**Purpose:**

The prospective, comparative evaluation of combined navigated laser photocoagulation and intravitreal ranibizumab in the treatment of diabetic macular edema has shown advantage of a combination therapy compared to ranibizumab monotherapy at year 1 with significantly reduced injections. The purpose of this retrospective study was to determine the long-term visual gains and need of injections in a 3 year-follow-up period.

**Methods:**

Retrospective analysis of patients of the original study in the long-term follow-up from month 12 to 36. BCVA measurements following the original 1 year study were taken using logMAR charts. Injections were provided with standard of care using PRN, based on change in BCVA and CRT using SD-OCT scans. Main outcome measures were change in BCVA and mean number of injections from 12 to 36 months.

**Results:**

BCVA was stable in both groups from 12 through 36 months, showing a change of 0.16 ± 0.1 log MAR. Following the initial reduction in required injections at month 12, combination therapy patients continued to require 1.3 times fewer injections over the next 24 months (2.91 ± 2.3 vs 3.85±3.7 injections for monotherapy).

**Conclusions:**

Combination of navigated laser and ranibizumab achieved BCVA gains equivalent to anti-VEGF monotherapy. These results could be maintained through month 36. Required injections were 2.0 injections lower in year 1 and further 1.3 times fewer in year 2 and 3 in the combination group compared to monotherapy. Adding navigated laser photocoagulation to intravitreal anti-VEGF therapy may still represent a superior therapeutic approach to DME patients.

## Introduction

Around 29% of patients with diabetes mellitus (DM) age 20 years or older will develop a diabetic macular edema (DME), and according to the WHO, DME is the leading cause of vision impairment in the working class. [1–3] Due to the success of antibody-derived inhibitors of vascular endothelial growth factor (VEGF), the management of DME has shifted to intravitreal injection therapy and has mostly replaced Macular Laser Therapy (MLT) as a first-line treatment.[4–7]

Pivotal anti-VEGF trials not only demonstrated a stabilisation of the disease in terms of reducing the central retinal thickness (CRT) using injection therapy, they also proved its safety and its ability to even improve patients´ visual acuity.[8]

To achieve these results however, adherence to a stringent regimen of frequent intravitreal injections—between seven and twelve in the first year—is required. [9–12]

From several real-life observations, we know that large numbers of intravitreal injections represent a high treatment burden for patients. Almost half of the diabetic patients would appreciate a reduction in the number of injections required to achieve the same objective outcomes in terms of visual acuity. [12] Additionally, for doctors and health care providers in an everyday clinical setting, treating every diabetic eye with intravitreal injections at the right time represents a substantial organisational challenge. [13, 14].

Conventional MLT monotherapy has been largely replaced by intravitreal injection therapy and several studies tried to evaluate the effect of laser treatment in combination with intravitreal injection therapy. [15] None of these trials demonstrated a significant effect on visual acuity or a significant reduction of intravitreal injections by adding conventional laser treatment however. [8, 10]

We and others have recently shown that the combination of intravitreal ranibizumab and navigated laser treatment in DME requires fewer injections over the course of the disease as compared to intravitreal anti-VEGF injection alone. [16]

Our own previous data on combining anti-VEGF therapy and MLT performed with the navigated laser (Navilas Laser System, OD-OS GmbH, Teltow, Germany) providing digital planning, image guidance and higher accuracy in laser spot application [17], demonstrated a significant reduction of anti-VEGF injections in the first year with in-trend better visual outcomes than ranibizumab monotherapy. [18]

Additionally, a minimized treatment burden through injection therapy with comparable visual outcomes remains a priority for many of our diabetic patients. [12]

Therefore, the aim of our retrospective analysis was to evaluate if the benefits of combination therapy, specifically the reduced number of anti-VEGF injections and comparable visual outcomes, could be maintained in a long-term follow-up period.

## Material and methods

The prior comparative evaluation of combined navigated laser photocoagulation and intravitreal ranibizumab in the treatment of diabetic macular edema was a prospective comparison study over 12 months with a total of 66 patients with center-involving DME.

Patients were enrolled between 2011 to 2012 at the Department of Ophthalmology at Ludwig-Maximilians-University, Munich, Germany. Inclusion criteria were male or female patients (aged>18 years) with center-involving DME in spectral domain optic coherence tomography (Spectralis OCT, Heidelberg Engineering GmbH, Heidelberg, Germany) secondary to diabetes mellitus type 1 or 2.

Patients were randomly assigned to two groups, patients in the monotherapy arm received an intravitreal injection therapy (IVT) with three injections of ranibizumab 0,5 mg (Rbz) followed by further injections given on a pro re nata therapy scheme (PRN scheme). The combination therapy group received additionally to ranibizumab 0,5 mg a navigated MLT (Spot size 100μm, pulse duration 100ms, mean number of spots 32,88, range 8–81, mean energy 95,18mJ, range 69-121mJ) after the third injection at month three, if the central retinal thickness (CRT) decreased to 445 μm or below. In case CRT was still above 445 μm an additional intravitreal injection was delivered and navigated MLT was performed four weeks afterwards. In the further course of the study intravitreal injections were delivered also in a PRN scheme.

On each visit the best corrected visual acuity (BCVA) and central retinal thickening (CRT) measured by spectral domain OCT (SD-OCT) was documented. The clinical examination and interpretation of the clinical appearance was performed by a retina specialist.

The current study is a retrospective analysis of 24 eyes of 22 patients of the original study who had a full follow-up for a minimum of 36 months.

Main outcome measures were the change in BCVA and CRT, measured in SD-OCT as well as the number of injections required in each of the follow up years as well as the total number of injections over the full course of this study.

Approval for this study was provided by the Institutional Review Board of the University Eye Hospital of Munich and adhered to the tenets of the Declaration of Helsinki.

All participants provided verbal informed consent to have data from their medical records used in research.

### Statistical methods

All data were collected in a MS-Excel 2013 spreadsheet (Microsoft Corporation, Redmond, WA) and analysed using the Statistical Package for Social Sciences version 24 for Windows (IBM, New York, USA). To test for differences in both groups, the non-parametric Mann Whitney U test was used.

Because of a loss of the majority of study subjects and lack of follow up data, we abstained from detailed statistical analysis.

## Results

Of the 66 original patients included in the one-year-data, 11 eyes of 11 patients in the combination group and 13 eyes of 11 patients in the ranibizumab monotherapy group were available to be included in the long term follow up of 36 months.

Although all missing patients were contacted and offered a long-term follow-up visit, 23 patients of the combination group and 21 patients of the monotherapy group either could not be reached despite contact by telephone and letter (20 in the combination group, 19 in the monotherapy group), or refused a follow–up visit due to long travel distances to the clinic (3 versus 2 in the monotherapy group).

Baseline characteristics of those with a full follow up of 36 months were similar in both groups despite the reduced number of patients and are summarized in Table 1.

**Table 1:** Baseline characteristics of patients with completed 3 year follow up.

	Ranibizumab + Navilas MLT	Ranibizumab Monotherapy
Number of eyes	11	13
Mean Age	65.8 ± 9.1	67.4 ± 11.8
Baseline BCVA in log Mar	0.55 ± 0.3	0.58 ± 0.29
Baseline OCT in μm	446 ± 152	425 ± 112
Mean Follow Up time in month	54 ± 6	54 ± 10

Mean baseline BCVA in log MAR was 0.55 ± 0.3 in the combination group and 0.58 ± 0.29 in the monotherapy group. Mean baseline CRT values were 446 μm ± 152 in the combination group and 425μm ± 112 in the monotherapy group.

In the combination group were 6 male and 5 female patients with a mean age of 65.8 ± 9.1 years, just as the monotherapy group which also comprised 6 male and 5 female patients with a mean age of 67.4 ± 11.8 years.

### Visual acuity and CRT development

In the combination group (Ranibizumab + Navilas MLT) an overall mean visual gain of -0.16 ±0.2 log Mar (Median -0,1) was achieved from baseline to 36 months, whereas in the Ranibizumab monotherapy group the mean visual gain was -0,02 ±0,2 logMar (Median 0). In the follow-up period from 12–36 months, both cohorts showed stable BCVA with a small change of 0,04 ±0,2 in the combination group (Median 0) and 0,09 ±0,3 log Mar (Median +0,1) in the monotherapy group.

**Fig 1:**
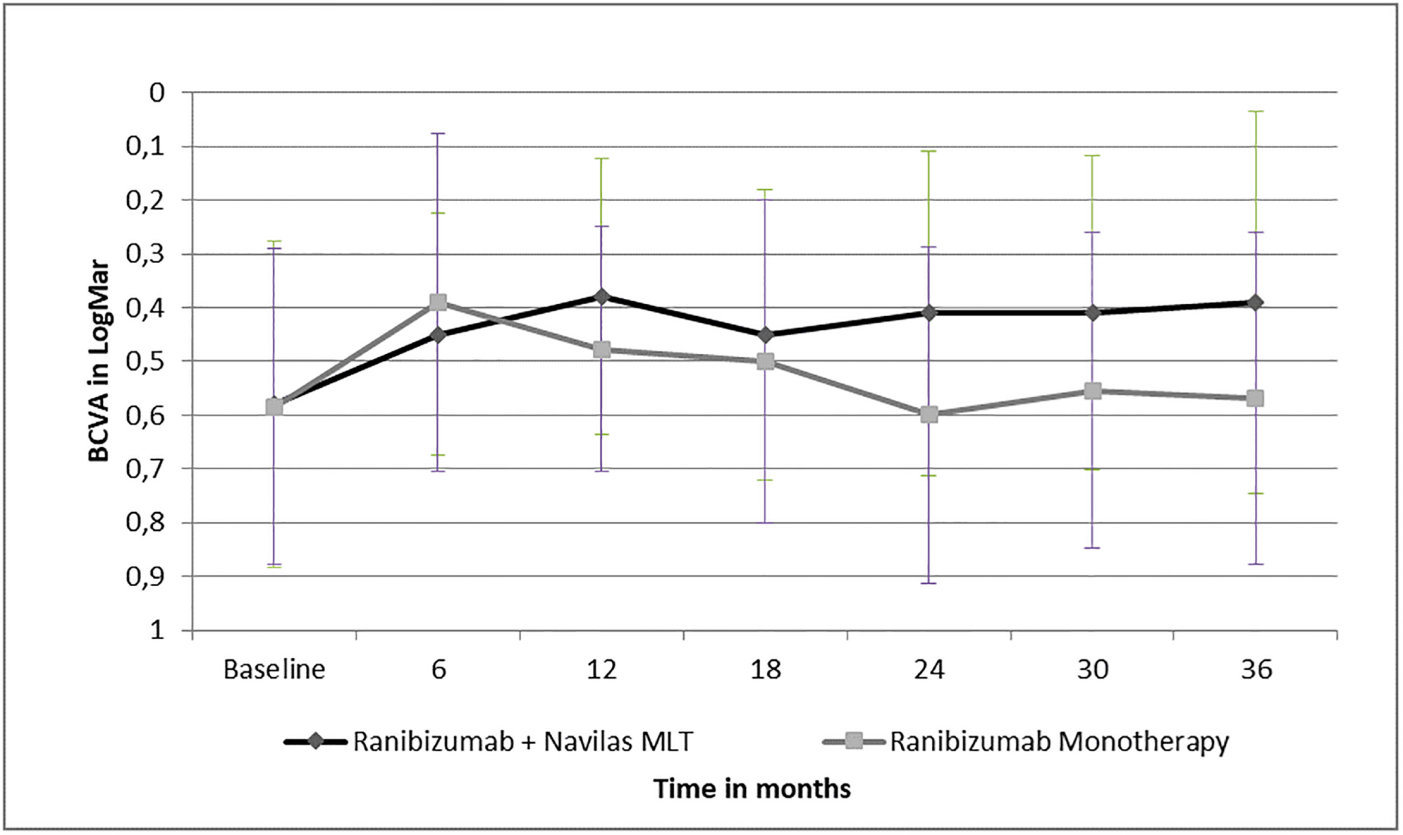
Best corrected visual acuity in log MAR from baseline to 36 months in both groups.

The overall better results regarding BCVA of the group with Navilas MLT of approximately 1 line was also maintained through month 36. (Fig 1)

There was no significant difference between the visual gain from baseline to month 36 (p = 0.067) and from month 12 to 36 (p = 0.299).

**Fig 2:**
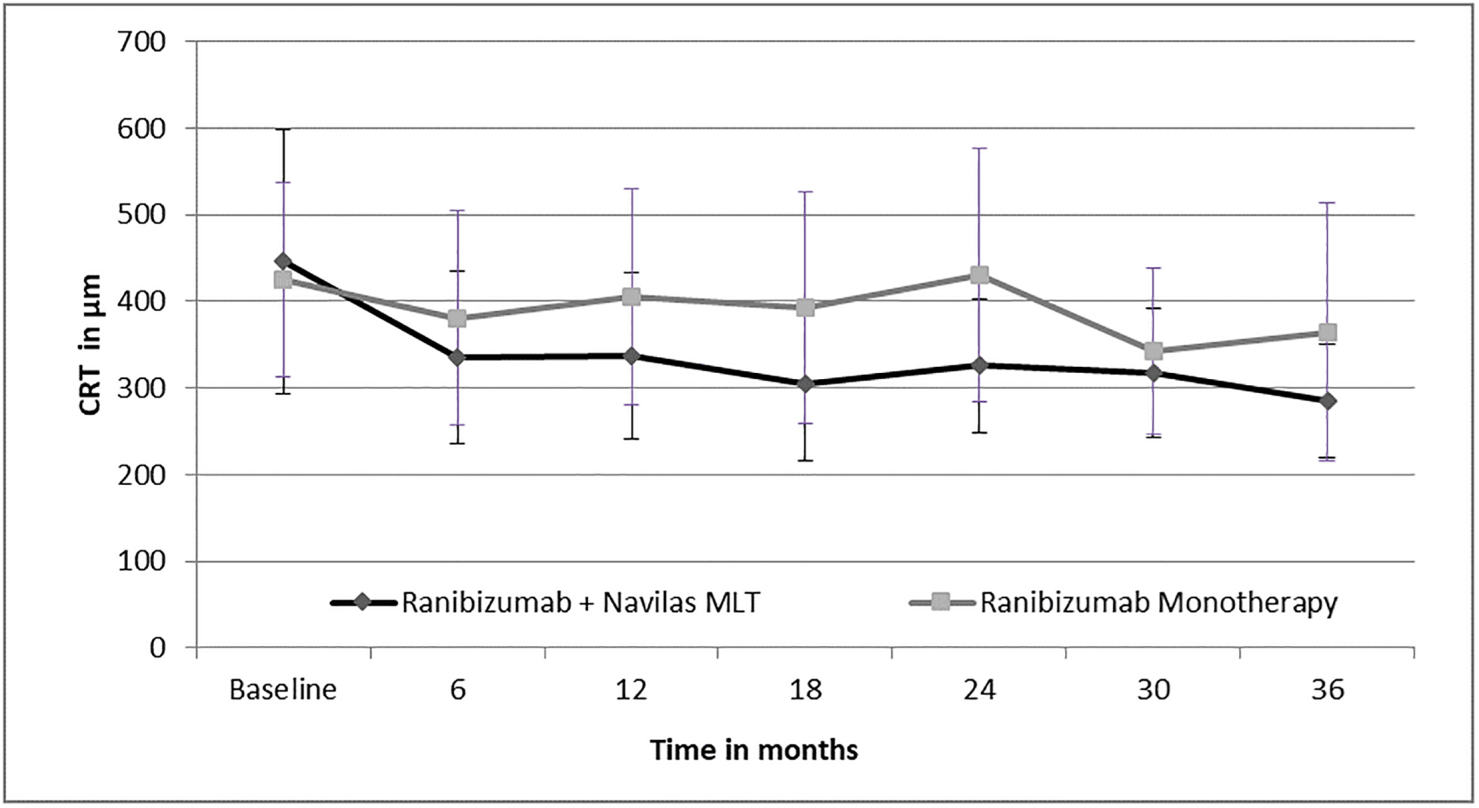
Reduction of central retinal thickness in μm from baseline to 36 months.

The mean overall reduction of CRT in the combination group was -161μm ± 184 (Median -131) from baseline to month 36 and -52μm ± 109 (Median -48) from months 12 to 36. Less mean overall reduction in CRT and also in the long term follow up from 12 to 36 months (-61 μm ± 206, Median -104 and -41μm ± 141, Median -41) was achieved in the monotherapy group. (Fig 2)

The difference between the groups was not statistically significant (p = 0.225) for CRT reduction from baseline to month 36 and for CRT reduction from month 12 to 36 (p = 0.829).

### Retreatment rate and number of injections

**Fig 3:**
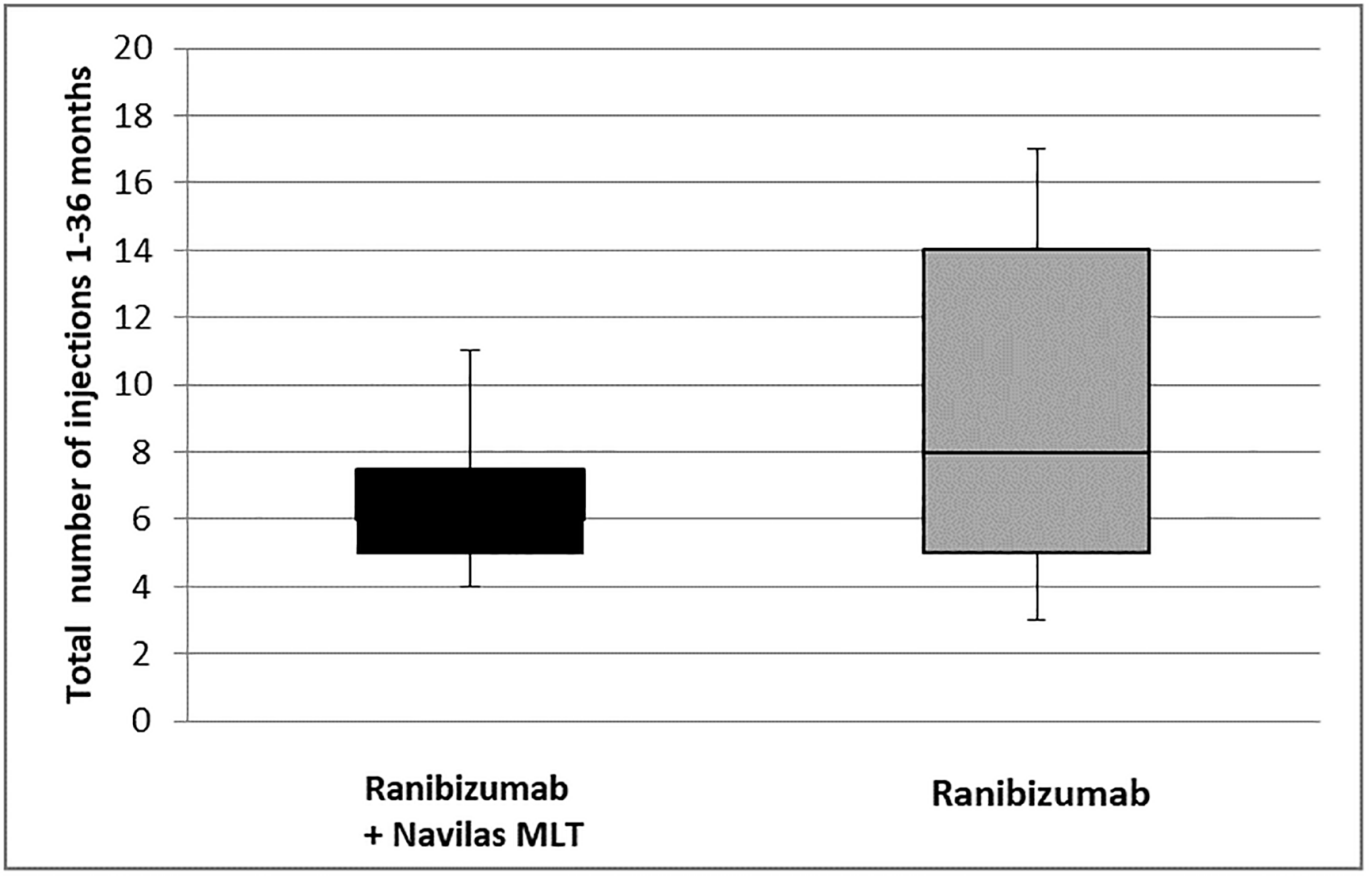
Total number of injections from month 1 to 36. Combination therapy shows a median of 6 injections within 36 month. Monotherapy shows a median of 8 injections within 36 months.

The long-term follow up from baseline to 36 months showed a lower total number of injections in the combination group (Mean 6.6 ± 2.5, Median 6.0) compared to the monotherapy group (9.3 ± 5.1, Median 8.0). (Fig 3)

**Fig 4:**
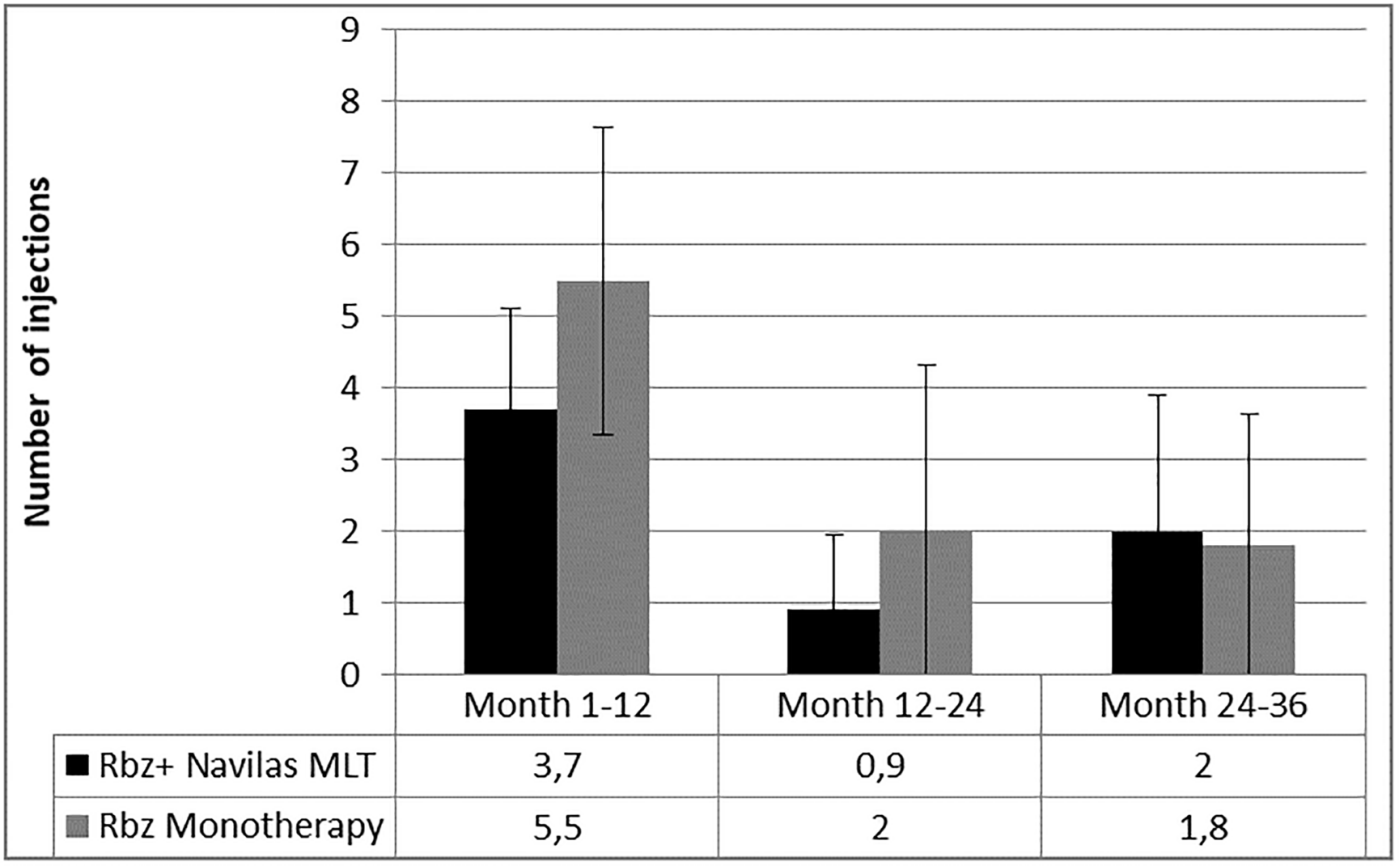
Number of injections in every year of the 36 month follow-up.

Following the initial reduction in required injections in the first 12 months, combination therapy patients also continued to require 1.3 times fewer injections over the next 24 months (2.91 ± 2.3 SD vs 3.85 ±3.8 SD injections for monotherapy). (Fig 4)

No significant difference between the number of injections from baseline to month 36 (p = 0.292) and from month 12 to 36 (p = 0.764) was observed.

In the combination therapy group, 3 eyes had consecutive laser re-treatment in the follow-up period from months 12 to 36 (27,3%).

No adverse effects of intravitreal injections or navigated laser were observed during the study in any of the patients.

## Discussion

It is apparent that the development of anti-VEGF intravitreal therapy has revolutionized the treatment of diabetic macular edema.

Randomized clinical trials such as RISE and RIDE proved the stabilizing and restoring effect of IVT on visual acuity for ranibizumab [4, 10], albeit with an injection frequency that requires a strictly organized treatment regimen provided by the physicians, and, importantly, good compliance and therapy adherence by the patients.

However, with around 382 million patients with diabetes worldwide in 2013, a number that is expected to rise to 592 million patients in 2035, [19] focus is shifting to provide a treatment regimen that is not only efficacious and safe, but also manageable under real-world conditions by both patients and doctors.

Our initial 12-month, prospective comparison of a standardized combination therapy regimen compared to an anti-VEGF monotherapy suggested a superiority in terms of re-treatment rate and overall injection burden in favour of the combination therapy. [18]

In this retrospective analysis of the patients with a 36-month long-term follow-up we have observed that the reduction in injection frequency of MLT and ranibizumab treated patients can be maintained over at least three years without inferior visual outcomes and with a similar reduction in CRT when compared to ranibizumab monotherapy. Indeed, the significant reduction of injections in year 1 was not only maintained but, although not statistically significant, further reduced by a factor of 1.3 in the combination group in years 2 and 3.

Nevertheless, we found a mild increase in injection rate only in the combination group in year 3 compared to year 2. In contrast to that, the overall injection rate in the first year in big clinical trials is with 7–9 injections higher than the injection rates reported in the 12-month data of our data. One could hypothesize that the comparable low injection rate in the first year, especially in the combination group did not calm down the whole dynamic process of DME in those patients and therefore resulted in that mild increase of injection rate in the third year.

Considering that in addition to the general disease burden, IVT is a crucial factor of diabetic patients´ quality of life, [12, 20] it is reasonable that the value and additional effect of macular laser treatment, either focal or grid, has continuously been investigated until now and controversies still exist. [15]

The recently published TREX-DME study compared monthly IVT injections with treat-and-extend with or without navigated laser photocoagulation. A significant difference in the number of injections between the monthly treated group and the treat-and-extend plus navigated laser group with fewer injections in the laser group was reported. Similar outcomes however, were seen at the one-year-time point in the treat-and-extent group regardless of additional navigated laser photocoagulation.[21]

Other studies on the other hand, such as READ-2, point to an additional benefit of MLT in reducing CRT. [5] Regarding the number of injections needed for comparable visual gains, several randomized controlled trials such as Protocol I, as well as several smaller studies, including ours, support the hypothesis that a reduction of required IVT can be achieved by combination therapy [4, 11]

While our 12 month data demonstrated a significant reduction in injections and a significantly lower retreatment rate in the combination group, there are several limitations to our retrospective analysis, largely owing to the small sample size.

First of all, due to a high loss of study subjects in the long term follow up possible significances and tendencies of the results have to be handled with care.

Furthermore we know from the 5-year-protocol I data that the biggest functional and anatomical gains can be achieved by deferring MLT by up to 6 months. [11]

Therefore study designs evaluating macular laser treatment and its additional therapeutic effect in combination with anti VEGF IVT might today differ from the design chosen in our 12 month data with comparable early laser intervention after 3–4 injections.

The fact that the overall reduction in injections, which was statistically significant in our 12-month data [18], is no longer significant in the long-term follow up of 36 months might be due to the smaller number of follow-up patients. This further supports the concern that adherence to a strict therapy regimen over such a long period of time is problematic, and that there might be a bias towards patients who have had a poorer course of the disease, with longer need for IVT.

While no consistent benefit from added conventional, non-navigated laser treatment to anti-VEGF treatment has been demonstrated up to now, the retrospective real-life observation of our DME patients after 3 years showed an overall lower injection burden in the combination therapy group with equivalent visual outcome.

Therefore, adding navigated macular laser treatment to a consequent intravitreal anti-VEGF therapy may still represent a reasonable therapeutic approach to a proportion DME patients with stable functional and anatomical results in the long term.

Larger and longer randomized trials with standardized, transparent laser recommendations concerning detailed laser parameters, exact laser strategies und when to perform within ongoing IVT—and in comparison to new subthreshold laser treatment regimen—may be helpful to define the remaining role of laser in times of pharmacotherapy for DME treatment.

## Supporting information

S1 Table Anonymized Dataset. (XLSX)
